# New evidence of gender inequality during COVID-19 outbreak in the Middle East and North Africa

**DOI:** 10.1016/j.heliyon.2023.e17705

**Published:** 2023-06-29

**Authors:** Suzan Abdel-Rahman, Fuad A. Awwad, Muhammad Qasim, Mohamed R. Abonazel

**Affiliations:** a Department of Demography and Biostatistics, Faculty of Graduate Studies for Statistical Research, Cairo University, Giza, Egypt; b Department of Quantitative Analysis, College of Business Administration, King Saud University, P.O. Box 71115, Riyadh, 11587, Saudi Arabia; c Department of Economics, Finance and Statistics, Jönköping University, Jönköping, Sweden; d Department of Applied Statistics and Econometrics, Faculty of Graduate Studies for Statistical Research, Cairo University, Giza, Egypt

**Keywords:** Employment outcomes, Gender gap, Job loss, Income reductions, MENA region, Multivariate probit model

## Abstract

The COVID-19 pandemic has significantly altered employment and income distribution, impacting women and men differently. This study investigates the negative effects of COVID-19 on the labour market, focusing on the gender gap in five countries in the Middle East and North Africa (MENA) region. The study indicates whether women are more susceptible to losing their jobs, either temporarily or permanently, switching their primary occupation, and experiencing decreased working hours and income compared to men during the COVID-19 outbreak. The study utilizes a multivariate Probit model to estimate the relationship between gender and adverse labour outcomes controlling for correlations among outcomes. Data are obtained from the Combined COVID-19 MENA Monitor Household Survey, covering Egypt, Tunisia, Morocco, Jordan, and Sudan. The findings of this study offer empirical evidence of the gender gap in labour market outcomes during the pandemic. Women are more likely than men to experience negative work outcomes, such as permanent job loss and change in their main job. The increased childcare and housework responsibilities have significantly impacted women's labour market outcomes during the pandemic. However, the availability of telework has reduced the likelihood of job loss among women. The study's results contribute to a better understanding of the impact of COVID-19 on gender inequality in understudied MENA countries. Mitigation policies should focus on supporting vulnerable women who have experienced disproportionate negative effects of COVID-19.

## 1 Introduction

COVID-19 has disrupted the global economy and caused widespread losses of income and livelihood in many countries. Large disparities have been found in labour market outcomes across countries during the pandemic [[1], [2], [3], [4]]. Less-educated workers, low-paid workers, youth, and working women have been severely affected by the COVID-19 pandemic [[5], [6], [7], [8]]. Workers in the informal sector have been hardest hit by the COVID-19 crisis and lost their jobs and livelihoods, especially those lacking insurance coverage and pensions. In contrast, workers with permanent contracts, who were paid regularly and had fixed working hours, were less likely to lose their jobs. Private companies laid off many workers, reduced nominal wages, and omitted regular wage increases, especially during the first months of the stringent lockdown period [9]. College-educated, white-collar, and high-income workers were able to continue their work from home compared with less-educated, blue-collar, and low-income earners who had to stay in the workplace or lose their jobs [10,11].

Although previous recessions affected the livelihoods of males more than females, the COVID-19 pandemic has caused disproportionate impacts on jobs and incomes, exacerbating pre-existing gender gap in labour outcomes [[12], [13], [14], [15]]. On the supply side, restrictions imposed by the pandemic (e.g., social distancing, closure of schools, and suspension of childcare homes) have directly affected women's employment, especially working mothers. Working mothers were more likely to lose their jobs due to increased childcare and housework responsibilities [16]. On the demand side, women are more concentrated in service sectors and industries that require high physical proximity and were therefore severely affected by the pandemic [17,18]. Inflexible conditions during the pandemic worsened women's economic outcomes, and in general, women in both paid and unpaid work were more likely to lose their jobs and incomes than males [[19], [20], [21]].

Gender inequality is alarming in less developed countries, especially in Arab countries. Women are mistreated in their work life due to difficulties in obtaining a job, adequate income, and promotions like their male counterparts [22]. Women are also abused in family life in terms of physical and sexual violence from partners [23,24]. Despite increasing efforts to empower women and enhance their role in MENA labour market, the economic downturn caused by COVID-19 has destabilized women's employment. Lockdown measures and restriction of teleworking have undermined Arab women's careers. As a result, women experienced negative labour outcomes including job losses and substantial reductions in working hours and incomes [25,26].

Considering the peculiarity of the MENA region, two points are particularly relevant for the region. First, women have substantially lower employment and participation rates, despite their high educational attainment and other socioeconomic markers, The Gender Development Index in the Arab region (0.856) is lower than the global average (0.941) [27]. Women's participation in the Arab labour market is weak, with unemployment rate reaching 19% compared with 8% for men in Arab countries in 2019. According to the 2020 World Economic Forum's Gender Gap Report, Tunisia, Egypt, Jordan, and Morocco rank 124,134,138, and 143, respectively, out of 153 countries on the Gender Gap Index, and rank 141, 143,138, and 14, respectively, in labour force participation rate, while ratios of female to male labour force participation rate equal 0.36,0.32,0.22, and 0.31, respectively, indicating significant gender gap in labour force participation rates in Arab countries [28]. Second, a high concentration of employed women was noted in labour-intensive sectors, with a large percentage working in the informal sector (61.8%) in Arab countries.

A growing number of studies have investigated the implications of COVID-19 on gender inequality worldwide. Some studies measured the gender impacts of COVID-19 on income and employment outcomes in a local context, such as Spain [19], the USA(3,15,18,21), and the UK [17]. Other studies focused on exploring gender differences across countries [1,2,4]. In contrast, limited studies were conducted in the MENA region due to the pandemic's novelty and data availability challenges. Barsoum and Majbouri [29] focused on the impact of COVID-19 on work, care work, and subjective well-being with a particular focus on women in the MENA region. Ramadan [30] measured determinants of household income decline in Jordan and demonstrated that informal workers, youth, women, workers in hard-hit sectors, and poor households are more likely to lose their incomes. In a descriptive study, ElBehairy et al. [31] overviewed the macroeconomic impacts of COVID-19 and compared unemployment and participation rates before and after the pandemic, finding that females in the private sector were mostly affected by the COVID-19 outbreak. In a recent policy brief, Krafft et al. [32,33] described the impact of the COVID-19 pandemic on participation and employment rates in Arab countries. They summarized the fiscal, monetary, and social protection policies during the pandemic. Most COVID-19 studies in the MENA region typically described labour market evolution and induced changes in participation and employment rates during the pandemic. These studies have a particular focus on policy responses; apart from their focus on policy responses, their analyses and findings have tended to be qualitative [25,26,[31], [32], [33]].

Afrin and Shammi [34] reviewed how the women in Bangladesh are negatively exposed to the COVID-19 pandemic in terms of labour market contribution, employment loss, and health and wellness. Also, Fayyad and Al-Senawy [35] examined the legality of actions taken by employers against workers in Palestine throughout the COVID-19 pandemic.

To the best of our knowledge, this study is one of the few studies that provide empirical evidence on the gendered impacts of COVID-19 in the MENA labour market. Despite the prevalence of multiple negative work outcomes during the COVID-19 pandemic, including job and income losses, changes in main activities, and reductions in working hours and hourly wages, no prior investigation considered all these outcomes by gender over the countries we analyze. This study contributes to previous literature in several ways. First, the study investigates the implications of COVID-19 crisis on gender inequality in labour market outcomes in the under-studied MENA region. Second, the study extends the conventional analysis of factors affecting labour market outcomes. We used the multivariate probit model (MPM) to measure the determinants of experiencing multiple negative labour outcomes considering the potential correlations between outcomes. Previous studies were limited to bivariate outcomes and assumed unrealistic independence. Third, the study quantifies the role of increased childcare and housework responsibilities in shaping women's labour market outcomes during the pandemic and highlights how new work arrangements, such as remote work technology, have succeeded in retaining jobs and income during the pandemic.

### 1.1 Theoretical framework and study hypotheses

After a careful review of previous literature, we drew up the conceptual framework and identified the most important drivers of gender gap in labour market outcomes during the pandemic. The coronavirus and associated precautionary measures have affected the gender gap in labour outcomes across multiple dynamics:

- **Care responsibilities** have significantly altered women's work patterns and widened the gender gap in many countries [1,20]. Women are challenged by the burden of childcare, housework, and caring for elderly and sick household members during the pandemic. The presence of young children limited women's ability to participate in the labour market during the pandemic [21,36]. Previous studies demonstrated that women lost their jobs and incomes due to caregiving and housework responsibilities [4,17,37,38]. On the other hand, some studies argue that, in contrast to previous recessions, the COVID-19 crisis has affected women's work patterns differently. Layoffs and reduced working days minimized the gender childcare gap, allowing childcare responsibilities to be largely shared between parents [16,20,39,40]. Childcare responsibilities shifted to fathers when working mothers had to work outside the home [17]. However, women are the main responsible for childcare and housework tasks in Arab countries. Social norms prevailing in Arab countries encourage fathers to leave core caregiving and homeschooling responsibilities to women. In this context, we hypothesize that the increased women's responsibilities during the pandemic have substantially affected their jobs.
- **Socioeconomic status** illustrates disparities in labour market outcomes during the pandemic. Highly educated workers were more likely to work from home or be furloughed during the pandemic than less educated workers who had to work outside their homes or lose their jobs [8,20]. The economic effects of the pandemic have disproportionately affected all households across different income levels. Lower-income workers were more likely to suffer income drops and negative labour outcomes than higher-income workers [3,4,8].
- **Demographic characteristics** explain the disproportionate incurring of the negative repercussions of the pandemic. Married women have a heightened risk of losing their jobs during the pandemic outbreak, especially since childcare responsibilities are not evenly distributed among parents. Never-married women spent more time in paid work than their ever-married counterparts [37]. Dubois found that family characteristics such as marriage and the presence of children are associated with lower rates of women's inactivity. Place of residence reflects the heterogeneity in participation rates and labour market conditions across areas. Moreover, there are differences in virus spread and containment measures across areas. Urban workers have struggled with lockdown measures due to suspension of most economic activities concentrated in urban areas. Age reflects cumulative experience, which could raise the probability of maintaining jobs even during the pandemic outbreak. Béland et al. [41], Crowley et al. [42], and Mamgain [43] found that younger workers are more vulnerable to disruptions associated with the COVID-19 pandemic and suffered relatively more job losses than others.
- **Pre-pandemic work characteristics** are associated with labour market outcomes during the pandemic. The private sector, informal, irregular, and uninsured workers were the most vulnerable to income and job losses during the pandemic [25,26,32,33]. Therefore, we controlled the work characteristics while measuring the gender gap in labour outcomes. Work characteristics include occupation, economic activity, sector, work stability, health insurance coverage, and work inside the establishment. Telework is a potential determinant of the gender gap in labour outcomes during the pandemic. Workers who could not perform their tasks from home also experienced job losses and income fall [8,10,11].

This study focuses on the gender inequality in experiencing job loss (temporarily or permanently), reduced working hours and income, and changes in main jobs during the pandemic. The study hypothesizes that self-reporting of these outcomes is correlated, for example, reporting permanent layoffs or reduced working hours is expected to be highly correlated with income fall. Therefore, we used the multivariate Probit model to control correlations among outcomes while modeling the relationships between gender and adverse labour outcomes.

To meet the study objectives, the study is organized into six sections. After the introduction, Section 2 presents the data source. Section 3 illustrates the applied model and statistical methodology. Section 4 describes the induced changes in the labour market by the pandemic, measures the gender gap in labour outcomes, and provides the empirical results of the multivariate probit models. Finally, section 5 discusses the results, and Section 6 presents the conclusion.

## 2 Method and Materials

### 2.1 Data source

The study draws on the Combined COVID-19 MENA Monitor Household Survey (CCMMHH) conducted by the Economic Research Forum (ERF). The CCMMHH survey comprised harmonized data from Egypt (*N* = 3923), Tunisia (*N* = 6134), Morocco (*N* = 6144), Jordan (*N* = 2549), and Sudan (*N* = 2400). CCMMHH covered a national random sample of mobile phone users aged 18–64 and integrated data through three rounds (November 2020, February 2021, and April 2021). In November, the baseline wave was collected for Tunisia, Morocco, and Egypt. The second wave was collected in February 2021 for Tunisia, Morocco, Egypt, and Jordan. The third wave was collected in April 2021 for Tunisia, Morocco, Jordan, and Sudan. CCMMHH are repeated cross-sectional surveys that permit the study of the consequences of the pandemic over time. CCMMHH is constructed using phone surveys to indicate the socioeconomic and labour market impact of the global COVID-19 pandemic on households. It includes several sections covering demographic and household characteristics, education, labour market status, income, social safety net, employment and unemployment detection, employment characteristics, and social distancing. The survey has many features. First, it documents detailed information on labour outcomes, work arrangements, and work history before and after the COVID-19 pandemic, allowing the investigation of the various changes in employment and incomes. Second, the survey provides harmonized data that facilitates the comparison of COVID-19 consequences across countries and waves. Third, it allows for further study of the impacts of the pandemic on vulnerable groups, including informal and irregular workers, low-skilled workers, women, and youth. The survey collected labour market outcomes and work characteristics for wage workers. The analysis is limited to workers employed before the COVID-19 outbreak (February 2020). The individual weights are used during analysis because the outcome variables are at the individual level. For more details about the sampling procedure, weighting, attrition, and response rates across countries and waves, please, refer to Ref. [44].

### 2.2 Statistical analysis

Binary logistic regression, the most widely used model, is inefficient since the relationships between response variables are not utilized. Multinominal models also suffer from the same problem because they assume the independence of irrelevant alternatives. The multivariate probit model (MPM) provides a valuable alternative because it fully exploits the correction structure. MPM is a generalization of the probit model used to model multiple outcomes simultaneously. MPM is more appropriate for jointly predicting several outcomes on an individual-specific basis [45]. Therefore, the MPM is employed to model correlated labour market outcomes. MPM is characterized by the parameters' interpretability and flexibility in manipulating correlation structures. The model takes the following form:

$$y_{im}^{*}=\beta_{m}^{\prime }X_{im}+\varepsilon_{im},i=1,\ldots ,N\;and\;m=1,\ldots ,M$$

$$y_{im}=\left\{ \begin{array}{c} 1\;if\;y_{im}^{*}>0\\ 0\;otherwise \end{array}\right.$$

$y_{im}$ is the dependent variable with m binary response components. i refers to the worker, and m denotes the employment outcomes (temporary layoff/suspension, permanent layoff/suspension, decrease in working hours, decrease in income, change in the main job). $y_{im}$ takes the value one if worker i has experienced the outcome m and zero otherwise. β is the p×M coefficient matrix. X is the (n×p) design matrix of all independent variables. N and p are the numbers of observations and predictors. $\varepsilon_{im}$ are the error terms distributed as multivariate normal, with zero mean and variance-covariance matrix V. V has values of 1 on the main diagonal, and correlations $\rho_{jk}=\rho_{kj}$ on the off-diagonal elements [44]. It's worth labour market outcomes ($y_{im}$) are non-mutually exclusive choices and worker can mention more than one outcome at the same time.

The log-likelihood function takes the form:

$$L=\sum_{i=1}^{N}\omega_{i}{\log\;\Phi }_{5}\left(\mu_{i};\Omega \right)$$

$\omega_{i}$ is the weight for sample observation i=1,…,N. We used the individual weights in all analyses because the outcome variables are at the individual level. $\Phi_{5}\left(.\right)$ is the multivariate standard normal distribution with arguments ($\mu_{i};\Omega$) where:

$$\mu_{i}=\left(k_{i1}\beta_{1}^{\prime }X_{i1},k_{i2}\beta_{2}^{\prime }X_{i2},k_{i3}\beta_{3}^{\prime }X_{i3},k_{i4}\beta_{4}^{\prime }X_{i4},k_{i5}\beta_{5}^{\prime }X_{i5}\right)$$

with $k_{ik}=2y_{ik}-1$ for each i,k=1,2,…,5. Matrix Ω has elements $\Omega_{jk}$, where $\Omega_{jj}=1$ for j=1,…,5 , $\Omega_{21}=\Omega_{12}=k_{i1}k_{i2}\rho_{21}$ , …, $\Omega_{54}=\Omega_{45}=k_{i5}k_{i4}\rho_{54}$.

We estimated the 5-equation multivariate probit model using simulated maximum likelihood (SML). SLM yields relatively efficient and better approximations than standard linear numerical approximations. SLM estimator is consistent, asymptotic, and more efficient. The simulated probabilities are unbiased and bounded within the [0.1] interval. There are different simulation methods for evaluating the multivariate normal distribution function $\Phi_{m}\left(.\right)$ such as Stern, acceptance-rejection, and Geweke-Hajivassiliour-Keane (GHK) simulators. We adopted the GHK simulator, the most used simulator, due to its relatively high efficiency. For more details, see Ref. [46]. We used the Stata program “mvprobit” to fit the multivariate probit models using the GHK simulation method for the maximum likelihood estimation. The MPM of dimension m consists of m marginal probability distributions. Each distribution corresponds to a specific outcome, and m(m−1)/2 correlations indicate the association between the occurrence of the m outcomes. If the correlations differ from zero, the multivariate probability distributions are required to produce the probabilities of all outcomes’ combinations. We test the significance of correlations between errors terms (rho) using the likelihood ratio test, where the null hypothesis ($H_{0}$) is $H_{0}\;:\;\rho_{21}=\rho_{31}=\rho_{41}=\rho_{51}=\rho_{31}=\rho_{41}=\rho_{51}=\rho_{32}=\rho_{42}=\rho_{52}=\rho_{43}=\rho_{53}=\rho_{54}=0$, which means that there are no correlations between error terms of the five equations (labour market outcomes). Rejecting the null hypothesis justifies the use of the multivariate probit model.

Labour market outcomes are regressed on the gender variable while controlling for sociodemographic variables and pre-COVID-19 work characteristics. Sociodemographic variables included age, place of residence, marital status, education level, household size, and income quartile. The model also controlled waves and countries using fixed effects. The retrospective data on employment status in February 2020 allows us to measure induced changes in the labour market during the COVID-19 pandemic across countries and waves. Interactions between telework availability and female dummy were included in the model. Table 1 presents women's responsibilities and other control variables. To put a special focus on the gender gap and delve into gender issues, we added interaction terms between female and caregiving responsibilities to the models, which enables us to measure the impact of increased demand for childcare and housework on incurring negative work outcomes.

**Table 1:** Information about the variables used in the analysis.

Variables	Description and coding
Dependent variables	
Experiencing temporary layoff/suspension Experiencing permanent layoff/suspension Experiencing a decrease in working hours Experiencing a decrease in income Experiencing a change in the main job	Workers are asked if they have experienced these negative changes due to coronavirus or related restrictions in the last 60 days. They are dichotomous variables taking 1 if the worker experienced the outcome and 0 otherwise.
**Independent variables**	
**Sociodemographic variables** Age	Continuous variable. The survey targeted respondents between 18 and 64.
Gender	Binary variable takes 1 if female and 0 if male.
Place of residence	Binary variable takes1 if urban and 0 if rural.
Household size	Count variable indicates the number of household members including the children.
Marital status	Categorical variable consists of three categories: never married (reference group), currently married, and divorced/widowed. We convert it to a binary variable with 1 for currently married workers and 0 otherwise
Highest level of education	Categorical variable reflects the highest educational level completed by the worker: less than basic (reference group), basic education, secondary education, and higher education.
Income quartile	Categorical variable reflects household income quartiles. The first quartile is the reference group.
**Job characteristics before COVID-19**	
Main economic activity	Categorical variable describes the worker's main job/activity as of the end of February 2020. It consists of eleven broad categories: agriculture, fishing or mining, manufacturing, construction or utilities, retail or wholesale, transportation and storage, accommodation and food services, information and communication, financial activities or real estate, education, health, other services.
Occupation	Categorical variable describes the worker's occupation. It includes four broad categories: blue collar, skilled agricultural, production &transport, clerks/service workers, technicians/associate professionals, and manager/professional.
Employment stability	Binary variable refers to the stability of the employment status, takes 1 if the worker is engaged in regular (permanent or temporary) and 0 in the case of irregular work (causal, seasonal, or intermittent)
Type of sector	Binary variable indicates the type of sector, taking 1 for public sector workers and 0 for private sector workers.
Social insurance	Binary variable indicates social insurance status, taking 1 for insured workers and 0 for uninsured workers.
Working inside establishment	Binary variable takes 1 for workers inside establishment and 0 otherwise
**Factors associated with the pandemic**	
Able to work from home	Binary variable takes 1 if the worker was able to work from home and zero otherwise.
Caregiving and housework responsibilities *	
**Wom1**: Having children under age six	Binary variable indicates the presence of children under age six in the household
**Wom2**: number of children who are enrolled in school	Count variable indicates the number of children enrolled in school and living, in their current household.
**Wom3:** Childcare hours	Count variable indicates the number of hours a woman spent caring for children on a typical day in the pandemic period.
**Wom4**: Amount of time spent caring for children	Women were asked to compare the time spent in childcare in the past week compared to a normal week before the pandemic on a scale of 3 (more than usual, the same, and less than usual). We convert it to a binary variable taking 1 for more than usual category and 0 otherwise.
**Wom5**: Housework hours	count variable indicates the number of hours a woman spent doing housework on a typical day in the pandemic period.
**Wom6:** Amount of time spent doing housework	Women were asked to compare the time spent in childcare in the past week compared to a normal week before the pandemic on a scale of 3 (more than usual, the same, and less than usual). We convert it to a binary variable taking 1 for more than usual category and 0 otherwise.

*Questions about caregiving responsibilities were only asked to women having children. Therefore, they enter the model through using interaction terms with female variable. Childcare and housework questions were asked in Tunisia and Morocco in the three waves. while these variables where not included in the country-level analysis in Egypt, Sudan, and Jordan to avoid data loss as they were asked only in the second wave.

We checked the data and the validity of the variables' values. Cook's distance and standardized residuals are measured to check the influential values. There are no influential observations as the absolute standardized residuals are less than 3. To avoid the effect of multicollinearity on the magnitude of standard errors and to ensure the accuracy of coefficient estimates, the variance inflation factor (VIF) is computed. VIF measures how much the variance of a regression coefficient is inflated due to multicollinearity. As a rule of thumb, if the VIF value exceeds 5 that indicates a problematic amount of collinearity [47]. The value of VIF of all variables did not exceed 5, showing moderate correlations.

## 3 Results

### 3.1 COVID-19 induced labour market outcomes

COVID-19-induced labour market outcomes included permanent and temporary layoffs, changes in economic activities, and reduced working hours accompanied by income losses. According to the results, the work arrangements for 52.9% of workers have changed due to the COVID-19 outbreak. 13.7% of the respondents changed their main job/activity, while 86.3% maintained the same activity during the pandemic. Among workers engaged in formal work before the pandemic, 20% experienced temporary layoffs/suspension without pay while 9.32% were permanently laid off/suspended during the pandemic. In addition, 17% of workers saw their hourly wages drop, 21.1% suffered delays in wage payments, and 11.42% reported that their income stayed the same during the pandemic.

**Fig. 1:**
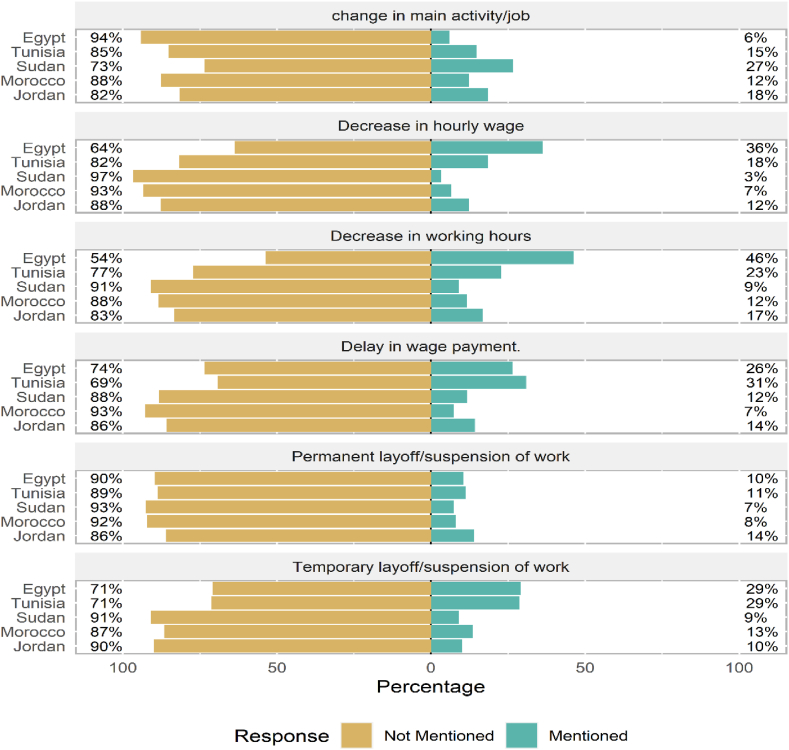
COVID-19 induced labour market outcomes across countries. **Note:** Respondents are asked whether they have experienced temporary layoff/suspension of work (without pay), permanent layoff/suspension of work, change in the number of working hours, and change in hourly wage in the last 60 days due to Covid-19 or its related restrictions.

There were notable differences in labour market outcomes between countries. As shown in Fig. 1, Egypt and Tunisia recorded the highest incidence of temporary layoffs with equal proportions (29%) compared to 13% in Morocco, 10% in Jordan, and 9% in Sudan. Tunisia and Egypt also recorded the highest proportions of workers who witnessed reductions in working hours and incomes; 36% and 18% of Egyptian and Tunisian workers mentioned income drops, and 46% and 23% saw their working hours reduced, respectively. The highest incidence of permanent layoffs was recorded among Jordanian workers (14%), while the lowest was among Sudanese workers (7%). In addition, 27% of Sudanese workers had to change their jobs versus 15% and 6% of workers in Tunisia and Egypt, respectively.

**Fig. 2:**
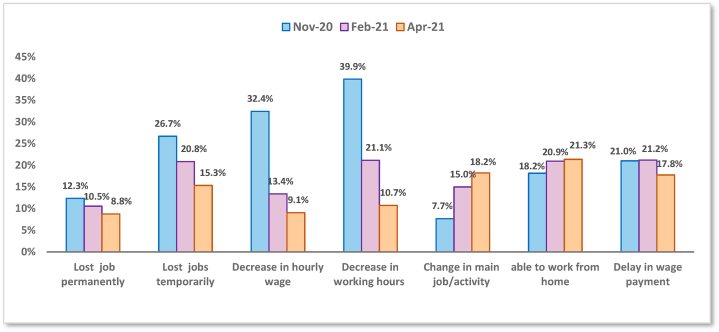
The incidence rate of different labour market outcomes across waves.

Over time, most countries have taken various policies to contain the virus and buffer its negative effects on the labour market. Flexible work arrangements have been adopted to continue the workflow and prevent workers from suffering financially. These include working remotely, shift patterns, reduced working hours, paid sick leave, and childcare leave for pregnant working mothers and those with children under 18. In response to these policies, negative labour market outcomes have diminished. As depicted in Fig. 2, the proportions of wage workers who experienced adverse labour outcomes have decreased across the waves. The proportion of workers reporting reduced incomes dropped considerably from 32.4% in November 2020 (wave 1) to 9.05% in April 2021 (wave 3). The proportion of temporarily laid-off workers also dropped from 26.7% in wave 1–15.3% in wave 3. The corresponding figures for permanently laid-off workers were 12.3% and 8.8%, respectively. Interestingly, there was a noticeable difference in the ability to work from home across the waves, with 21.3% of workers becoming able to work from home in the last wave compared to 18.2% in the first wave. The striking findings are the increased proportion of workers who have changed their main activity across waves, indicating their increased mobility across economic activities to secure their income.

**Fig. 3:**
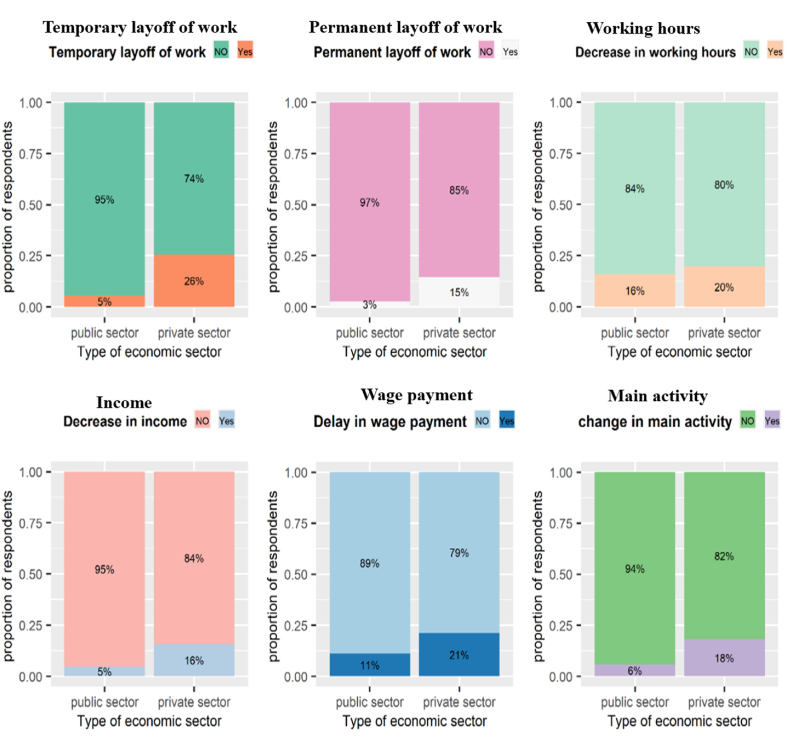
COVID-19-induced labour market outcomes by the economic sector.

The most interesting aspect of Fig. 3 is the government/public sector's success in mitigating the negative effects of the pandemic on labour market outcomes compared with the private sector. Private sector workers are the hardest hit by the negative effects of the pandemic recession. The proportion of permanently laid-off workers in the private sector is five times that of the government sector, more than a quarter of private sector workers were temporarily laid off, 21% experienced wage delays, and 20% had to work fewer hours. On the contrary, public-sector workers have maintained their job with infinitesimal changes in their incomes and economic activities. Only 5% of public-sector workers faced a decrease in income, and 6% had to change their main activity compared with 16% and 18% in the private sector, respectively. Private sector companies have resorted to layoffs and salary cuts to reduce financial losses caused by the COVID-19 recession. Many respondents attributed the change in their employment status to business status, and the challenges business owners face. About 34.4% of private-sector workers, compared to 18.6% of public-sector workers, mentioned that their companies had difficulty accessing customers due to government-imposed mobility restrictions. Also, 44% and 15% of private and public sector workers reported that the companies confronted a significant drop in demand for many reasons (e.g., regular customers could no longer afford their products or services and cancelled orders).

### 3.2 Gender gap in labour market outcomes

The COVID-19 pandemic has affected males and females differently. As summarized in Table 2, job losses and income fall were more prevalent among males than females, 21.5% of males reported temporary job loss versus 18% of females, and 20.1% of males experienced a decline in their hourly wages versus 14.8 of females. Also, there are notable differences in work arrangements during the COVID-19 outbreak in favour of women; 34.6% of women versus only 16% of men were able to work from home. In addition, 25.2% of women versus 24.6% of men have experienced a reduction in working hours. However, a high proportion of females had to change their main job/activity during the COVID-19 outbreak (17.4% of females versus 11.1% of males). In addition, the gender differences in the proportion of permanently laid-off workers are insignificant.

**Table 2:** Proportion of workers reporting labour market outcomes by gender.

Labour market changes caused by COVID-19	Female	Male	Difference and 95% Confidence interval [CI]
Workers lost their jobs permanently	0.102	0.106	- 0.004 [-0.022,0.014]
Workers lost their jobs temporarily	0.180	0.215	−0.035** [-0.058, −0.011]
Workers experienced a decrease in hourly wage	0.148	0.201	−0.053*** [-0.074, −0.032]
Workers had to change their main job/activity	0.174	0.111	0.063*** [0.039, 0.085]
Workers were able to work from home	0.346	0.160	0.186*** [0.159, 0.212]
Workers experienced a decrease in working hours	0.246	0.252	−0006* [-0.031, 0.018]
Workers experienced stability in their incomes	0.829	0.773	0.056*** [0.035, 0.078]
Workers experienced a delay in wage payment	0.197	0.203	−0.006 [-0.029, 0.018]

*** p<0.001, ** p<0.01, * p<0.05.

**Fig. 4:**
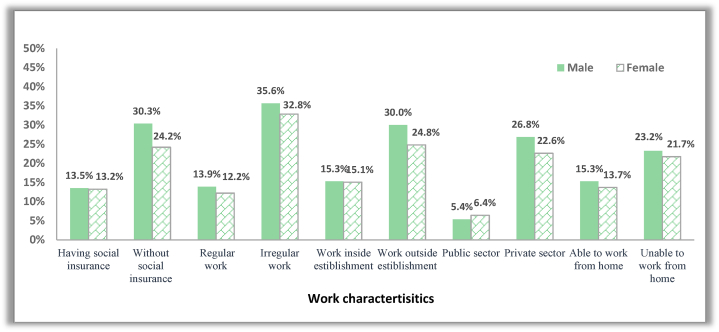
The incidence rate of temporary job loss among males and females across different work characteristics.

**Fig. 5:**
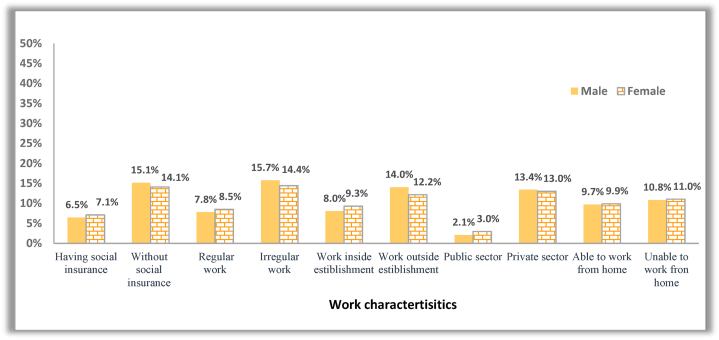
The incidence rate of permanent job loss between males and females across different job characteristics.

**Note:** In the CCMMHH survey, workers were asked about their work characteristics before COVID-19 (in February 2020), which include social insurance, employment stability, type of economic sector (public, private), work inside establishments, and whether they were in regular work (permanent or temporary) or irregular work (causal, seasonal, or intermittent). They also asked about the ability to work from home during the COVID-19 outbreak.

Workers experienced disproportionate labour market disruptions according to their work characteristics before the pandemic. Job losses were more pronounced among the informal, private sector, uninsured and non-institutional workers (Fig. 4). Irregular workers were more likely to lose their jobs; 35.6% and 32.8% of males and females in irregular jobs reported temporary job loss compared to 13.9% and 12.2 in regular jobs. Social insurance played a protection tool against job loss; about 13.5% and 13.2% of insured males and females, respectively, suffered temporary job loss compared to 30.3% and 24.2% of their uninsured peers. Substantial differences were found between the public and private sectors, with 26.8% and 22.6% of males and females in the private sector lost their jobs temporarily, compared with 5.4% and 6.4% of males and females in the public sector. The same conclusions can be drawn for the effect of work characteristics on permanent job loss (Fig. 5). It is apparent that men were more negatively affected than females, and temporary job loss was more prevalent among males than females in all work characteristics except for the public sector. While women were more exposed than males to permanent job loss under certain job characteristics. Women working in regular work, in the public sector, and inside establishments are more likely to be permanently laid off than males. Therefore, women's work characteristics should be controlled to reveal the actual gender gap in the negative work outcomes triggered by the pandemic.

**Fig. 6:**
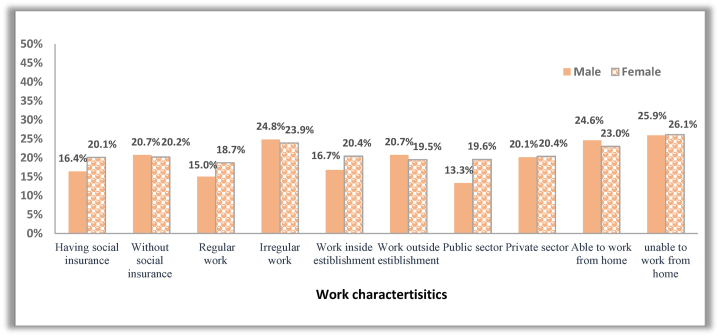
Incidence rate of working reduced hours among males and females across different work characteristics.

**Fig. 7:**
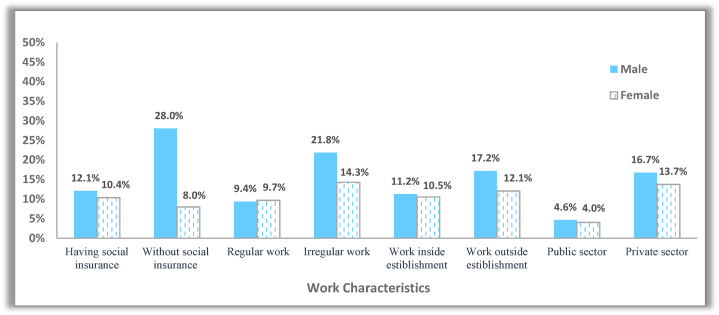
Incidence rate of experiencing a decrease in wage payment among males and females across different work characteristics.

**Fig. 8:**
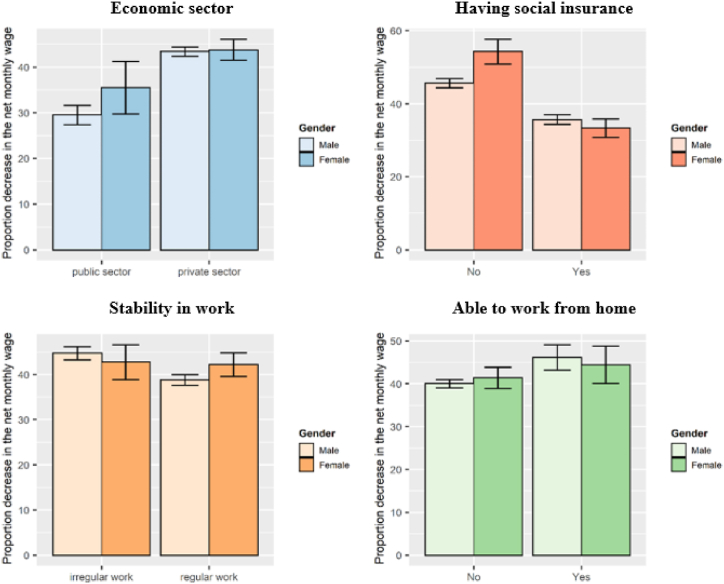
Mean and 95% CI of proportion decrease in net monthly wage by work arrangements.

Working hours have differed markedly by gender and work characteristics. As shown in Fig. 6, women working in the public sector, in regular work and with social insurance, have enjoyed reduced working hours more than males in the same work arrangement. However, men were more likely to have wage cuts. Fig. 7 shows that 12.1% of insured males and 4.6% of males in the public sector experienced decrease in wages compared with 10.4% and 4% of females, respectively. Moreover, income reductions were more pronounced among males in disadvantaged work conditions. The proportion of uninsured males whose wages declined was three and a half times that of females. The corresponding figures for irregular work were 21.8% for males versus 14.3% for females. Fig. 8 displays the percentage decrease in monthly wage by gender and work characteristics. Private sector, irregular, and uninsured workers suffered the largest income decreases, with an average decrease of more than 40%. However, safe working conditions (working in the government sector and having regular work) did not protect women from declining wages more than males. Also, women who could not work from home and lacked social insurance experienced a higher drop in income than males.

**Fig. 9:**
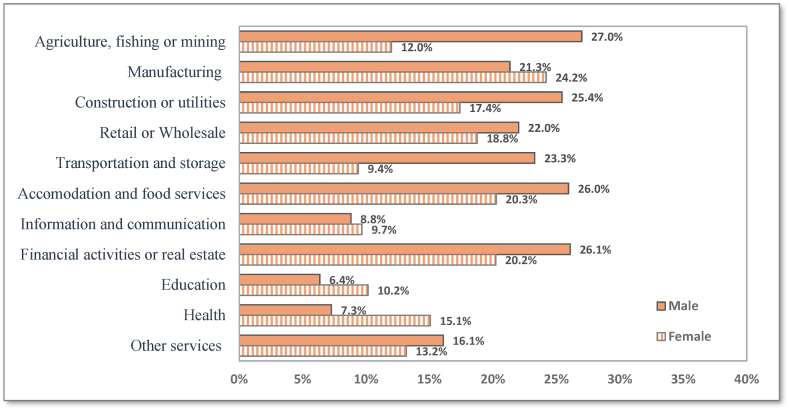
Incidence rate of decrease in hourly wage among males and females in different jobs/activities.

**Fig. 10:**
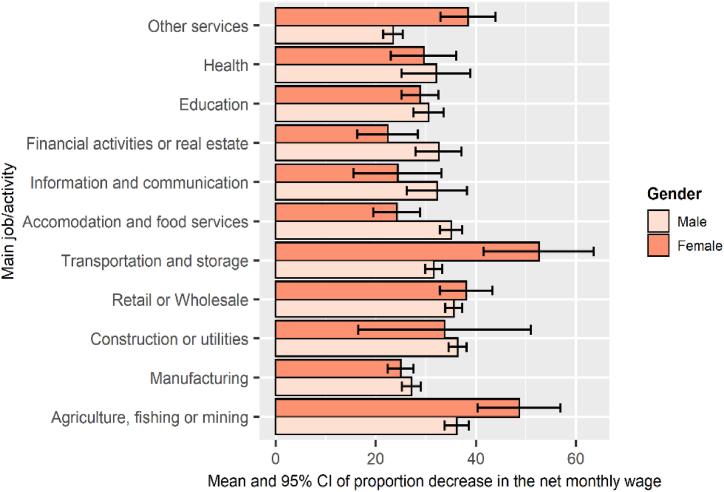
Mean and 95% CI of proportion decrease in net monthly wage by economic activity and gender.

Income reductions differed across economic activities. A breakdown of incidence rates of income decline by economic activity and gender is shown in Fig. 9. Males were more affected in the following major activity groups: agriculture, fishing or mining, financial activities and real estate, and accommodation and food services industries than their counterparts in other jobs/activities. Among males, the highest incidence rate of income decline (27%) was recorded in the agriculture, fishing or mining activities, while the lowest rate was in the educational activities (6.4%). Regarding the gender gap, the proportion of males who have endured income decline is more than twice that of females in agriculture and finishing activities (27% versus 12%) and around one and half that of females in construction and utilities industry (25.4% versus 17.4). In contrast, the incidence rates of income reduction among females in health, education, manufacturing, and Information and communication activities exceeded those for males by 7.8%,3.8%, 2.9%, and 0.9%, respectively. Fig. 10 displays the percentage decrease in monthly wages disaggregated by gender and economic activity. Males' incomes in most economic activities decreased by 30%–40%, while the decrease in females’ incomes exceeded 40% in some activities. As depicted in Fig. 10, the percentage decrease is greater for women than for males in transportation and storage, agriculture, and finishing, and retail or wholesale trade.

**Fig. 11:**
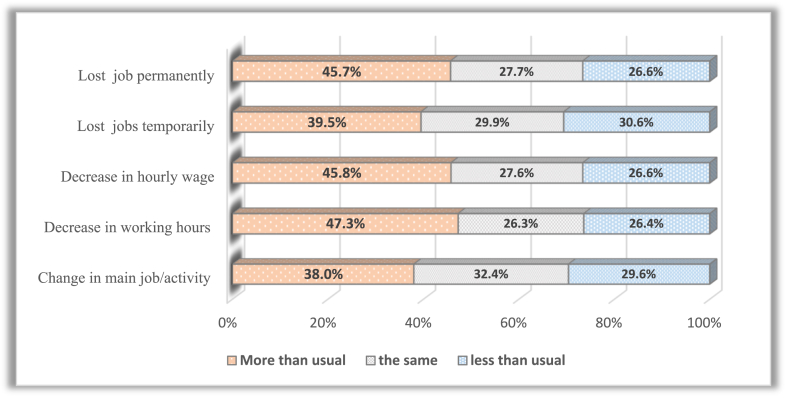
Distribution of negative labour outcomes among women according to the change in time spent caring for children before and after COVID-19.

**Fig. 12:**
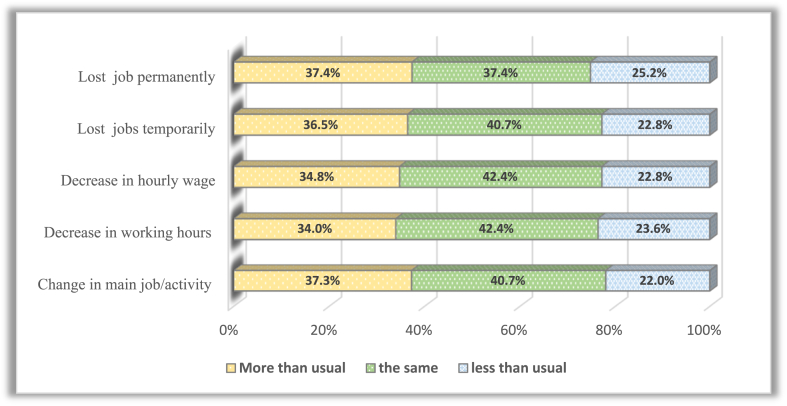
Distribution of negative labour outcomes among women according to the change in time spent on housework before and after COVID-19.

Increased care responsibilities during the COVID-19 outbreak significantly affected labour market outcomes. Childcare and household chores increased during the pandemic compared to February 2020 (before the pandemic). Significant differences were found between the time allocated to childcare and housework before and after the pandemic. In addition, 37.7% of women devoted more time to childcare than usual before the pandemic, and 39.5% reported an increase in childcare time compared to the school closure period. Furthermore, 34.3% of women spent more time on housework after COVID-19. Negative labour outcomes are more prevalent among women who spent more time caring for children during the pandemic (Fig. 11). In comparison, most women who suffered negative labour outcomes spent the same time as before the pandemic on housework (Fig. 12).

**Fig. 13:**
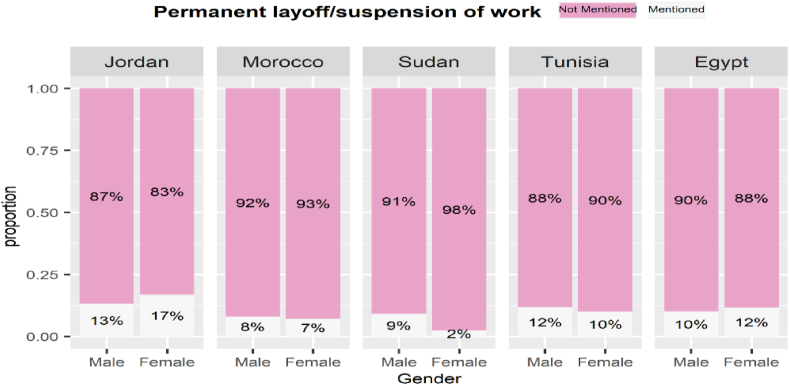
The gender gap in permanent layoffs by country.

**Fig. 14:**
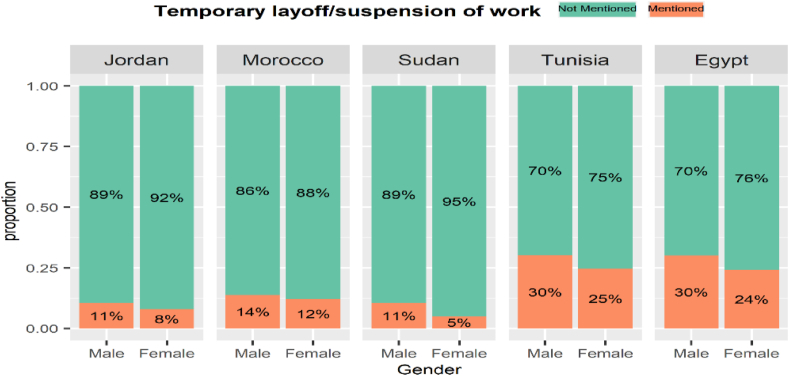
The gender gap in temporary layoffs by country.

**Fig. 15:**
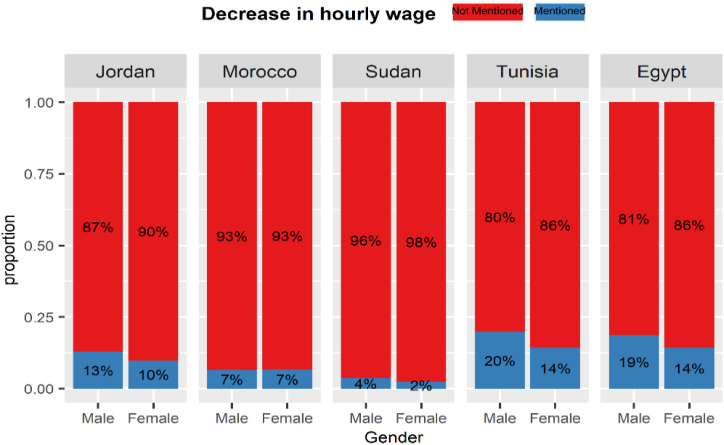
The gender gap in income losses by country.

**Fig. 16:**
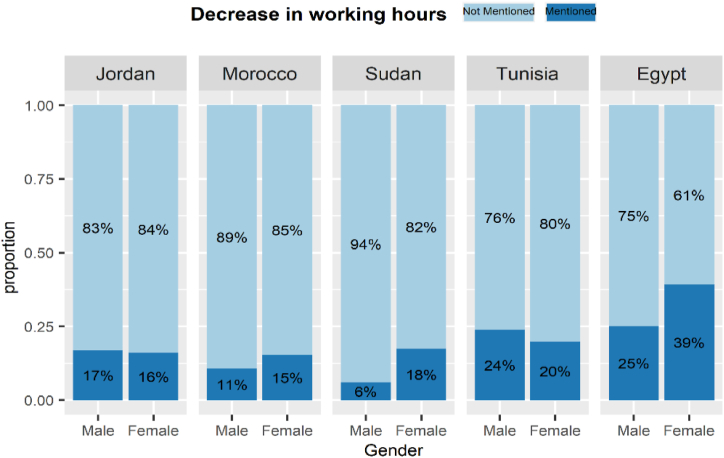
The gender gap in reduced working hours by country.

The gender impact of COVID-19 on the labour market varied across MENA countries. Fig. 13, Fig. 14, Fig. 15, Fig. 16 display labour market outcomes by gender in the five countries. In both Jordan and Egypt, the gender gap in permanent layoffs was not in favour of females. The proportion of females who lost their jobs permanently was 17% compared to 13% for males in Jordan, and 12% versus 10% for males in Egypt. However, permanent layoffs were more prevalent among males than females in other countries. Also, the proportions of males who have been temporarily laid off are higher than females in all countries. A similar conclusion was found for income decline, where the proportions of males reporting reduced incomes are higher than females in most countries. In contrast, females in Morocco, Sudan, and Egypt had reduced working hours more than males, particularly in Egypt, where 39% of females reported reduced working hours due to COVID-19 workplace restrictions versus 25% of males.

### 3.3 Determinants of COVID-19 labour market outcome

**Fig. 17:**
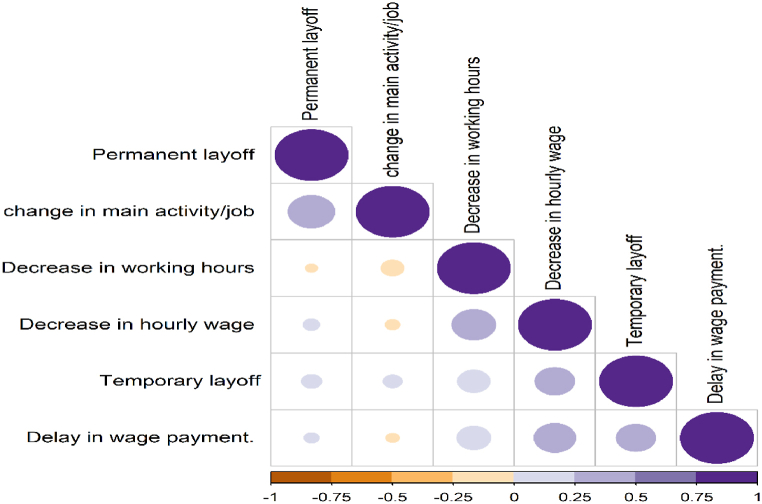
Correlogram plot of correlation between labour market outcomes during the COVID-19 pandemic. **Note**: Positive and negative correlations are displayed in purple and brown. Both Color intensity and circle size are proportional to the correlation coefficients. (For interpretation of the references to colour in this figure legend, the reader is referred to the Web version of this article.)

Workers reported multiple labour outcomes (non-mutually exclusive choices). The correlogram plot, Fig. 17, indicates the strength of the association between outcomes. A delay in wage payment, in particular, is highly correlated with other outcomes. These correlations have been considered through a joint estimation of outcomes using the multivariate probit model (MPM) [48]. Table 3 shows the determinants of different labour market outcomes during the COVID-19 outbreak in MENA region. Table 3 provides the regression coefficient (Coeff) and standard error (SE). The gender gap is evident in some labour market outcomes in favour of males. As indicated in MPM results, women were more likely to be permanently laid off and change their main activity than males across MENA countries. Women also experienced a significant reduction in working hours.

**Table 3:** Determinants of labour market outcomes during COVID-19 in MENA countries.

Variables	Temporary layoff/suspension	Permanent layoff/suspension	Decrease in working hours	Decrease in income	Change in the main job
	Coeff (SE)	Coeff (SE)	Coeff (SE)	Coeff (SE)	Coeff (SE)
Age	−0.006*(0.003)	0.001 (0.002)	−0.003 (0.00)	−0.006*(0.003)	0.001 (0003)
**Female**	−0.008 (0.058)	0.136**(0.051)	0.124* (0.054)	−0.039(0.063)	0.309***(0.064)
Urban	0.145**(0.051)	−0.005 (0.058)	0.006 (0.049)	−0.067 (0.055)	0.005 (0.063)
Currently married.	0.03(0.054)	−0.133*(0.061)	0.002 (0.053)	0.078(0.059)	−0.241***(0.063)
**Highest level of education**					
Basic	−0.054 (0.067)	0.063 (0.076)	−0.015 (0.070)	−0.129 (0.075)	0.009 (0.084)
Secondary	−0.056 (0.062)	−0.036 (0.071)	0.066(0.064)	0.041 (0.067)	0.037 (0.078)
Higher education	−0.125*(0.072)	−0.029 (0.086)	0.084(0.075)	−0.012 (0.083)	0.053 (0.091)
**Income quartile**					
Second quartile	0.004(0.062)	0.008 (0.069)	0.068 (0.064)	0.094 (0.068)	−0.014 (0.073)
Third quartile	−0.153*(0.067)	−0.127*(0.075)	0.015 (0.068)	0.031(0.074)	−0.099 (0.081)
Fourth quartile	−0.359***(0.086)	−0.198*(0.096)	−0.115 (0.082)	−0.191*(0.093)	−0.143 (0.098)
**Household size**	0.018*(0.010)	0.0357***(0.011)	0.003(0.01)	0.001 (0.011)	0.032 ** (0.011)
**Interaction terms between female and care responsibilities during COVID-19**					
Having children under age six	0.027 (0.625)	0.106 (0.121)	0.204**(0.065)	0.042 (0.069)	−0.008 (0.066)
Number of children enrolled in school	0.541** (0.082)	0.041(0.045)	0.011 (0.040)	0.064 (0.048)	−0.035(0.044)
Childcare hours	0.009 (0.014)	0.027*(0.013)	0.024* (0.011)	0.005(0.016)	0.032*(0.014)
Housework hours	0.351* (0.051)	0.020 (0.024)	0.023 (0.021)	0.004 (0.025)	0.019(0.022)
Spending more time caring for children	0.279* (0.140)	0.157 (0.0157)	0.412**(0.132)	0.325**(0.110)	0.224 (0.147)
Spending more time doing housework	0.101 (0.125)	0.127 (0.149)	0.096 (0.134)	0.179*(0.089)	0.164 (0.139)
**Female able to work from home**	−0.038(0.058)	−0.150*(0.0651)	0.036 (0.053)	−0.059 (0.063)	0.175**(0.064)
**Work characteristics fixed effects**	**Yes**	**Yes**	**Yes**	**Yes**	**Yes**
**Occupation fixed effects**	**Yes**	**Yes**	**Yes**	**Yes**	**Yes**
**Economic activity fixed effects**	**Yes**	**Yes**	**Yes**	**Yes**	**Yes**
**Wave fixed effect**	**Yes**	**Yes**	**Yes**	**Yes**	**Yes**
**Country fixed effect**	**Yes**	**Yes**	**Yes**	**Yes**	**Yes**

*, **, *** signify the p-value <0.05, 0.01 and 0.001, respectively. Work characteristics include working in the government sector, regular work,and within the facility, in addition to health insurance coverage.

COVID-19-induced changes in care responsibilities significantly increased the risk of adverse labour market outcomes for women across MENA countries. The increased responsibilities of women clarify the channels through which the pandemic has affected the gender gap in MENA labour market. Spending more time caring for children during the pandemic compared to a normal week before the pandemic was positively correlated with temporary job losses and reduced working hours and income. While spending more time doing housework was correlated with income reduction. Women with children under age six were more likely to have reduced working hours. Women were more likely to lose work temporarily if the number of children enrolled in school increased. Increased childcare hours were positively associated with reduced working hours, permanent job loss, and major activity change. At the same time, increased housework hours were positively associated with temporary job loss. Workers who could work from home were less likely to be permanently laid off and more likely to change their primary activity.

Sociodemographic variables have limited effects on incurring negative work outcomes. Place of residence did not significantly affect labour outcomes except temporary job loss among urban workers. Increasing age reduced the likelihood of job losses and activity changes. Currently married workers are less subjected to permanent layoff/suspension and change of main activity. Highly educated workers were less likely to be laid off temporarily than less-educated workers. Similarly, workers in higher income quartiles were more protected from negative labour outcomes. Household size was positively correlated with the probability of facing negative work outcomes.

The determinants of labour market outcomes during the pandemic are separately estimated for each country, which allows capturing some interesting heterogeneity across countries. As indicated in Table 4, Table 5, Table 6, Table 7, Table 8, the gender gap persisted at the country level and was significant in one labour outcome at least. In Morocco and Egypt, women were more likely to change their main activities and experience reduced working hours. In Tunisia, women were more likely to lose their jobs permanently, experience reduced working hours, and change their main activities. In Jordan, women were more likely to lose their jobs permanently and change their main activities. In Sudan, women were more likely to have reduced working hours. Responsibilities affected women's labour market outcomes at the country level. Spending more time caring for children and doing household chores during the COVID-19 pandemic has been positively associated with temporary and permanent losses of jobs in Morocco. Spending more time doing housework also decreased women's incomes in Morocco. The presence of children less than six years increased the probability of changing main activities and permanent job loss for Tunisian women.

**Table 4:** Determinants of labour market outcomes during COVID-19 in Morocco.

Variables	Temporary layoff/suspension	Permanent layoff/suspension	Decrease in working hours	Decrease in income	Change in the main job
	Coeff (SE)	Coeff (SE)	Coeff (SE)	Coeff (SE)	Coeff (SE)
Age	−0.006 (0.004)	0.006(0.005)	−0.014**(0.005)	−0.006(0.006)	−0.007(0.005)
Female	0.026 (0.113)	0.064 (0.131)	0.227*(0.107)	0.096(0.133)	0.171*(0.074)
Urban	0.122(0.113)	−0.009(0.132)	0.046(0.116)	0.097(0.141)	−0.006(0.132)
Currently married	0.072(0.103)	−0.005 (0.121)	0.086(0.104)	−0.107(0.123)	−0.159(0.113)
**Highest level of education**					
Basic	−0.084(0.128)	0.091 (0.150)	0.203(0.137)	0.182(0.159)	0.116(0.154)
Secondary	−0.091(0.126)	−0.236 (0.151)	0.193(0.136)	0.085(0.162)	0.041(0.151)
Higher education	−0.016 (0.135)	−0.382* (0.161)	0.362*(0.144)	0.275(0.171)	0.142(0.159)
**Income quartile**					
Second quartile	0.036(0.109)	0.348*(0.141)	−0.185(0.114)	−0.120(0.134)	0.198(0.128)
Third quartile	−0.388*(0162)	0.162 (0.186)	−0.465**(0.155)	−0.239(0.184)	−0.147(0.176)
Fourth quartile	0.0242(0.195)	−0.113 (0.260)	−0.630**(0.218)	−0.331(0.254)	−0.167(0.232)
**Household size**	0.019(0.017)	0.016 (0.186)	0.005(0.018)	0.005(0.021)	0.027 (0.019)
**Interaction terms between female and responsibilities variables during COVID-19**					
Having children under age six	0.155(0.176)	0.167(0.222)	0.163 (0.159)	0.258(0.254)	0.019(0.18)
Number of children enrolled in school	0.003(0.111)	0.336*(0.122)	0.069(0.113)	−0.201(.191)	0.086(0.109)
Childcare hours	0.049(0.041)	0.003(0.050)	0.050(0.040)	0.101(0.064)	0.044(0.040)
Housework hours	0.002 (0.048)	0.042(0.063)	0.184(0.362)	0.185(0.011)	0.068(0.044)
Spending more time caring for children Spending more time doing housework	0.683***(0.101)	0.451*(0.212)	0.313(0.317)	0.248(0.191)	0.027(0.022)
	0.709*(0.312)	0.648*(0.269)	0.426(0.314)	1.063*(0.514)	0.005(0.023)
**Female able to work from home**	−0.126 (0.106)	−0.239*(0.088)	0.054 (0.102)	−0.076 (0.126)	0.026 (0.11)
**Work characteristics fixed effects**	**Yes**	**Yes**	**Yes**	**Yes**	**Yes**
**Occupation fixed effects**	**Yes**	**Yes**	**Yes**	**Yes**	**Yes**
**Economic activity fixed effects**	**Yes**	**Yes**	**Yes**	**Yes**	**Yes**
**Wave fixed effect**	**Yes**	**Yes**	**Yes**	**Yes**	**Yes**

*, **, *** signify the p-value <0.05, 0.01 and 0.001, respectively. Work characteristics include working in the government sector, regular work,and within the facility, in addition to health insurance coverage.

**Table 5:** Determinants of labour market outcomes during COVID-19 in Tunisia.

Variables	Temporary layoff/suspension	Permanent layoff/suspension	Decrease in working hours	Decrease in income	Change in the main job
	Coeff (SE)	Coeff (SE)	Coeff (SE)	Coeff (SE)	Coeff (SE)
Age	−0.015***(0.004)	−0.011*(0.005)	−0.004(0.003)	−0.011**(0.004)	−0.007(0.005)
Female	−0.096(0.076)	0.352***(0.105)	0.168*(0.077)	−0.153(0.085)	0.216*(105)
Urban	0.098(0.074)	0.019(0.092)	−0.067(0.076)	0.007(0.080)	0.079(0.116)
Currently married	−0.012(0.086)	−0.312*(0.0147)	−0.025(0.087)	0.018(0.094)	−0.404**(0.126)
**Highest level of education**					
Basic	−0.131(0.101)	−0.003(0.124)	0.001(0.011)	−0.112(0.110)	−0.226(0.158)
Secondary	−0.190*(0.089)	−0.005(0.119)	0.097(0.095)	0.041(0.096)	−0.184(0.138)
Higher education	−0.578***(0.112)	−0.285*(0.125)	−0.039(0.113)	−0.461***(0.124)	−0.312(0.168)
**Income quartile**					
Second quartile	−0.112(0.111)	−0.315*(0.127)	0.158(0.120)	0.027(0.119)	−0.514**(0.167)
Third quartile	−0.328**(0.107)	−0.533***(0.129)	−0.049(0.116)	−0.070(0.116)	−0.443**(0.154)
Fourth quartile	−0.897***(0.123)	−0.800***(0.146)	−0.111(0.127)	−0.504***(0.134)	−0.775***(0.179)
**Household size**	0.039*(0.018)	0.074***(0.022)	−0.025(0.087)	0.007(0.019)	0.066*(0.027)
**Interaction terms between female and responsibilities variables during COVID-19**					
Having children under age six	−0.065(0.096)	0.225*(0.111)	−0.231*(0.114)	0.101(0.104)	0.220*(0.111)
Number of children who are enrolled in school	−0.071(0.066)	0.033(0.081)	−0.008(0.072)	−0.068(0.076)	−0.117(0.094)
Childcare hours	0.020(0.025)	−0.009(0.032)	−0.022(0.028)	−0.012(0.029)	−0.022(0.035)
Housework hours	0.049(0.033)	0.103*(0.041)	−0.024(0.037)	−0.001(0.039)	0.068(0.045)
Spending more time caring for children Spending more time doing housework	0.075(0.197)	0.249(0.248)	0.306(0.205)	−0.174(0.226)	0.244(0.275)
	0.002(0.182)	0.157(0.242)	−0.076(0.194)	0.254(0.211)	0.332(0.262)
**Female able to work from home**	−0.113(0.191)	−0.220*(0.091)	0.0684(0.083)	−0.137(0.096)	0.144(0.114)
**Work characteristics fixed effects**	**Yes**	**Yes**	**Yes**	**Yes**	**Yes**
**Occupation fixed effects**	**Yes**	**Yes**	**Yes**	**Yes**	**Yes**
**Economic activity fixed effects**	**Yes**	**Yes**	**Yes**	**Yes**	**Yes**
**Wave fixed effect**	**Yes**	**Yes**	**Yes**	**Yes**	**Yes**

*, **, *** signify the p-value <0.05, 0.01 and 0.001, respectively. Work characteristics include working in the government sector, regular work,and within the facility, in addition to health insurance coverage.

**Table 6:** Determinants of labour market outcomes during COVID-19 in Egypt.

Variables	Temporary layoff/suspension	Permanent layoff/suspension	Decrease in working hours	Decrease in income	Change in the main job
	Coeff (SE)	Coeff (SE)	Coeff (SE)	Coeff (SE)	Coeff (SE)
Age	−0.015**(0.006)	0.011(0.007)	0.0002(0.005)	−0.005(0.006)	−0.005(0.007)
Female	−0.132(0.161)	0.053(0.173)	0.360**(0.0127)	−0.049(0.154)	0.566***(0.104)
Urban	0.229*(0.101)	−0.030(0.123)	0.113(0.094)	−0.377(0.206)	0.108(0.123)
Currently married	0.0905(0.130)	−0.304(0.160)	0.079(0.128)	0.323*(0.142)	−0.165(0.154)
**Highest level of education**					
Basic	0.009(0.181)	−0.095(0.231)	−0.096 (0.181)	−0.377(0.206)	−0.017(0.236)
Secondary	−0.117(0.144)	0.101(0.178)	−0.071(0.142)	0.010(0.151)	0125(0.186)
Higher education	−0.170(0.184)	−0.079(0.217)	0.002(0.164)	0.006(0.180)	0.309(0.226)
**Income quartile**					
Second quartile	−0.257(0.137)	−0.230(0.167)	0.155(0.135)	0.276(0.145)	−0.015(0.168)
Third quartile	−0.189(0.139)	−0.171(0.167)	0.159(0.136)	0.040(0.149)	−0.052(0.0173)
Fourth quartile	−0.588**(0.203)	−0.325(0.351)	0.197(0.182)	0.043(0.202)	0.016(0.226)
**Household size**	0.0359 (0.024)	0.025(0.029)	0.033(0.023)	0.006(0.026)	0.042 (0.030)
**Female able to work from home**	0.043(0.126)	−0.519***(0.130)	0.191* (0.107)	−0.121(0.127)	0.606***(0.122)
**Work characteristics fixed effects**	**Yes**	**Yes**	**Yes**	**Yes**	**Yes**
**Occupation fixed effects**	**Yes**	**Yes**	**Yes**	**Yes**	**Yes**
**Economic activity fixed effects**	**Yes**	**Yes**	**Yes**	**Yes**	**Yes**
**Wave fixed effect**	**Yes**	**Yes**	**Yes**	**Yes**	**Yes**

*, **, *** signify the p-value <0.05, 0.01 and 0.001, respectively. Work characteristics include working in the government sector, regular work,and within the facility, in addition to health insurance coverage.

**Table 7:** Determinants of labour market outcomes during COVID-19 in Jordan.

Variables	Temporary layoff/suspension	Permanent layoff/suspension	Decrease in working hours	Decrease in income	Change in the main job
	Coeff (SE)	Coeff (SE)	Coeff (SE)	Coeff (SE)	Coeff (SE)
Age	0.003(0.007)	0.006(0.006)	0.002(0.006)	0.002(0.006)	0.002(0.006)
Female	0.107(0.158)	0.331*(0.149)	−0.082(0.127)	−0.083(0.127)	0.378**(0.122)
Urban	−0.066(0.149)	0.064(0.159)	0.038(0.131)	0.038(0.132)	0.023(0.130)
Currently married	−0.005(0.157)	−0.075(0.146)	0.090(0.132)	0.090(0.132)	−0.416**(0.125)
**Highest level of education**					
Basic	−0.333*(0.153)	−0.026(0.195)	−0.467*(0.194)	−0.466*(0194)	−0.221(0.185)
Secondary	−0.492*(0.194)	−0.105(0.199)	−0.260(0.186)	−0.260(0.186)	−0.268(0.183)
Higher education	−0.679**(0.217)	−0.161(0.223)	−0.215(0.195)	−0.215(0.195)	−0.562**(0.197)
**Income quartile**					
Second quartile	−0.040(0.160)	−0.075(0.162)	0.135(0.151)	0.185(0.171)	−0.162(0.149)
Third quartile	−0.615**(0.198)	−0.131(0.182)	0.357*(0.199)	0.387*(0.179)	−0.160(0.162)
Fourth quartile	−0.993** (0.204)	−0.321(0.203)	0.153 (0.121)	0.173(0.192)	−0.200(0.172)
**Household size**	0.028 (0.028)	0.611*(0.310)	0.019(0.023)	0.0194(0.023)	0.013(0.002)
**Female able to work from home**	−0.046(0.165)	−0.201*(0.113)	0.088(0.114)	0.122(0.130)	0.176(0.119)
**Work characteristics fixed effects**	**Yes**	**Yes**	**Yes**	**Yes**	**Yes**
**Occupation fixed effects**	**Yes**	**Yes**	**Yes**	**Yes**	**Yes**
**Economic activity fixed effects**	**Yes**	**Yes**	**Yes**	**Yes**	**Yes**
**Wave fixed effect**	**Yes**	**Yes**	**Yes**	**Yes**	**Yes**

*, **, *** signify the p-value <0.05, 0.01 and 0.001, respectively. Work characteristics include working in the government sector, regular work,and within the facility, in addition to health insurance coverage.

Regarding the impact of demographic characteristics on labour market outcomes across countries, age is negatively associated with temporary layoffs in Egypt, reduced working hours in Morocco, change in main activity in Sudan and job loss and income reduction in Tunisia, while it had no significant effect on work outcomes in Jordan. Place of residence did not have a significant impact on labour market outcomes in countries except for Egypt and Sudan. Urban workers were more likely to lose their jobs temporarily in Egypt and change their economic activities in Sudan. The pandemic has exacerbated existing inequality between social groups in some MENA countries. Higher-income and higher-educated workers were less exposed to negative work outcomes than lower-income and less-educated workers, as in Jordan and Tunisia. As shown in Table 9, correlations between error terms (ρ) are significantly different from zero, indicating that the MPM is suitable for fitting data.

**Table 8:** Determinants of labour market outcomes during COVID-19 in Sudan.

Variables	Temporary layoff/suspension	Permanent layoff/suspension	Decrease in working hours	Decrease in income	Change in the main job
	Coeff (SE)	Coeff (SE)	Coeff (SE)	Coeff (SE)	Coeff (SE)
Age	−0.002(0.013)	−0.023(0.015)	−0.014(0.014)	−0.053(0.031)	−0.026*(0.010)
Female	−0.414(0.293)	−0.999(0.654)	0.530*(0.238)	−0.403(0.517)	−0.116 (0.202)
Urban	−0.007(0.365)	−0.389(0.375)	0.098(0.412)	−0.488(0.544	0.621*(0.316)
Currently married	−0.264(0.274)	0.327(0301)	0.208(0.290)	0.117(0.508)	−0.206(0.206)
**Highest level of education**					
Basic	0.214(0.543)	0.294(0.574)	−0.096(0.045)	−0.278(0.223)	−0.154(0.432)
Secondary	0.231(0.474)	0.257(0518)	−0.125(0.0115)	−0.385(0.286)	−0.280(0.365)
Higher education	−0.101(0.466)	0.035(0.514)	−0.253(0.198)	−0521(0.411)	−0.515(0.355)
**Income quartile**					
Second quartile	−0.432(0.423)	0.243(0.508)	0.382(0.532)	−0.258(0.214)	−0.753(0.347)
Third quartile	−0.723(0.450)	−0.292(0.551)	0.299 (0.566)	−0.385(0.312)	−0.595(0.338)
Fourth quartile	−0.178(0.376)	0.152(0.495)	0.114(0.523)	−0.526 (0.412)	−0.462 (0313)
**Household size**	0.003(0.037)	0.052(0.039)	−0.076(0.051)	0.142*(0.064)	−0.002(0.029)
**Female able to work from home**	−0.086(0.165)	0.151(0.133)	0.198(0.134)	0.172(0.140)	0.156(0.119)
**Work characteristics fixed effects**	**Yes**	**Yes**	**Yes**	**Yes**	**Yes**
**Occupation fixed effects**	**Yes**	**Yes**	**Yes**	**Yes**	**Yes**
**Economic activity fixed effects**	**Yes**	**Yes**	**Yes**	**Yes**	**Yes**
**Wave fixed effect**	**Yes**	**Yes**	**Yes**	**Yes**	**Yes**

*, **, *** signify the p-value <0.05, 0.01 and 0.001, respectively. Work characteristics include working in the government sector, regular work,and within the facility, in addition to health insurance coverage.

**Table 9:** Correlations between errors terms of labour market outcomes during COVID-19*.

	$\rho_{21}$	$\rho_{31}$	$\rho_{41}$	$\rho_{51}$	$\rho_{32}$	$\rho_{42}$	$\rho_{52}$	$\rho_{43}$	$\rho_{53}$	$\rho_{54}$	Likelihood ratio test^a^
Full model	0.004 (0.035)	0.219*** (0.030)	0.338*** (0.031)	0.081** (0.033)	−0.012** (0.034)	−0.024 (0.038)	0.553*** (0.026)	0.525 *** (0.255)	−0.218*** (0.034)	−0.158*** (0.036)	<0.001
Morocco	0.269*** (0.069)	0.087 (0.066)	0.133 (0.078)	0.190** (0.062)	−0.046 (0.079)	0.008 (0.088)	0.283 *** (0.066)	0.604*** (0.057)	−0.239** (0.073)	−0.171* (0.088)	<0.001
Tunisia	0.044 (0.066)	0.286 *** (0.057)	0.454 *** (0.057)	0.036 (0.063)	−0.013* (0.069)	0.006 (0.071)	0.677*** (0.042)	0.564*** (0.051)	−0.237** (0.070)	−0.035 (0.074)	<0.001
Egypt	−0.057 (0.071)	0.281 *** (0.057)	0.471 *** (0.0569)	0.085 (0.072)	0.017 (0.071)	−0.038 (0.080)	0.577*** (0.056)	00.517*** (0.052)	−0.184** (0.073)	0.006 (0.078)	<0.001
Jordan	−0.211** (0.080)	0.188 ** (0.075)	0.232 *** (0.073)	0.020 (0.785)	−0.384*** (0.074)	−0.516*** (0.079)	0.715*** (0.056)	0.610*** (0.053)	−0.437*** (0.716)	−0.531*** (0.069)	<0.001
Sudan	−0.196* (0.031)	0.198** (0.077)	0.251 ** (0.066)	0.282** (0.062)	0.027 (0.061)	0.385*** (0.070)	0.597*** (0.086)	0.681*** (0.072)	−0.098** (0.065)	0.321* (0.058)	<0.001

*Numbers 1 to 5 refer to temporary layoff/suspension, permanent layoff/suspension, decrease in working hours, decrease in income, and change in main job, respectively.

a The null hypothesis ($H_{0}$): $\rho_{21}=\rho_{31}=\rho_{41}=\rho_{51}=\rho_{31}=\rho_{41}=\rho_{51}=\rho_{32}=\rho_{42}=\rho_{52}=\rho_{43}=\rho_{53}=\rho_{54}=0$, which means that there are not correlations between error terms of the five equations (labour market outcomes).

## 4 Discussion

This study aims to document the adverse impact of the COVID-19 pandemic on labour market outcomes from a gender perspective, using the Combined COVID-19 MENA Monitor Household Survey. This study revealed a significant gender gap in labour market outcomes in MENA countries after controlling male-female differences in work characteristics and sociodemographic variables. Females were more vulnerable to negative work outcomes such as permanent layoffs and changing their main activity than males. This finding is consistent with previous studies, which found that the gender gap in the probability of job loss persisted even after controlling for work characteristics. Dang and Viet Nguyen [1] and Adams-Prassl et al. [4] found that women were more likely to lose their jobs permanently and experience income reductions than men. Piyapromdee and Spittal [6], Hupkau and Petrongolo [17], and Alon et al. [15] indicated that women were more likely to be laid off because they are over-represented in the major crowded industries that require workplace presence and have been disrupted by lockdown measures, such as the clothing industry, customer service companies, and retail and leisure industries. Bluedorn et al. [39] found that women's employment rate dropped proportionately more than men's rate in 25 countries in 2020. Tverdostup [36] and Reichelt et al. [49] also found that women were more negatively affected because they had to reduce their working hours and work remotely during the pandemic. On the other hand, some studies found contradictory results that females were less likely to lose their jobs than men [8] and the pandemic did not worsen the gender gap in employment outcomes [18,50].

Several factors explain the emerging gender gap in MENA labour market outcomes during the pandemic. Our results indicate that more than a quarter of women worked in educational services and 16% in manufacturing activities were severely affected by lockdown and social distancing measures. The incidence rates of income loss among women in education and manufacturing activities exceeded those of males by 3.8% and 2.9%, respectively. Women-dominated industries such as customer services, retail, home services, schools, childcare homes, and restaurants have been severely affected during the pandemic compared to the previous downturns in which male-dominated industries were hit hard [39].

Our results also indicate that care responsibilities significantly shaped women's labour outcomes during the pandemic. Spending more time caring for children during the pandemic positively correlated with temporary job loss and reduction in working hours. While spending more time doing housework was correlated with only income reduction. Women with children less than six years were more likely to have reduced working hours and more likely to lose work temporarily if the number of children enrolled in school increased. Our results are in line with the findings of Dang and Viet Nguyen [1], Adams-Prassl et al. [4], Sevilla et al. [20], Collins et al. [21], and Tverdostup [36] who indicated that childcare responsibilities caused significant changes in women's working patterns and increased gender gap in working hours. Sharing care responsibilities between parents does not apply to Arab countries, where childcare and housework fall on women's shoulders. Therefore, it was not surprising that working women with caregiving and housework responsibilities lost their jobs during the crisis.

Some individual characteristics significantly affected the likelihood of income and job losses. Consistent with the literature, our study found that education level was a significant determinant of work continuity during the COVID-19 pandemic, highly educated workers were more likely to maintain their jobs and incomes than less educated workers. Andrew et al. [16] also found that less-educated individuals were more likely to stop working partially or completely than those with a university degree. This result can be attributed to the highly educated workers concentrated in jobs which can be performed from home, in contrast to less-educated workers who are more prevalent in informal and physically demanding jobs. On the contrary, Adams-Prassl et al. [4] indicated that the effect of education was diminished after controlling for job characteristics.

Our study found that high-income workers were more protected against adverse labour market outcomes than low-income workers. This finding was reported in previous studies. Cajner et al. [3] and Blundell et al. [8] demonstrated that low-income workers were more likely to stop work and experience a significant reduction in earnings than high-income workers during the lockdown. Piyapromdee and Spittal [6] attributed this finding to that less educated and low-income workers are more likely to work in industries that require physical proximity and have experienced a marked decline in demand during the pandemic. Our study did not show that the worker's age and place of residence affected job loss and income drop. Galasso and Foucault also stated that no significant differences in labour market outcomes according to age groups were observed [2].

The negative effects of COVID-19 fade over time as labour market conditions improve across waves. Easing lockdown measures explains the noticeable improvement in work outcomes across the waves. In the beginning, due to the confirmed cases and increasing deaths, many companies implemented strict closures and reduced working hours. The negative repercussions of the closure measures on jobs and incomes forced companies to implement preventive measures less stringently than before. As a result, the labour market has continued, but with fewer hours, work shifts, and adopting remote work without layoffs. The companies provide workers with the required flexibility for business continuity. The proportion of workers who worked from home has increased, and those who reported reduced working hours and incomes have decreased across waves. Krafft et al. [25] reported that a small fraction of employed workers in Egypt, Tunisia, and Morocco was pushed out of the workforce in June 2021 compared to the beginning of the pandemic in February 2020 and previously unemployed individuals could enter the labour market. One of the important findings is that working from home significantly reduced the probability of losing jobs. Women who could work from home were less likely to be permanently laid off, while they were more likely to change their main activity. These findings align with previous studies that workers in remote-performing occupations are less likely to lose their jobs or experience reduced working hours compared to those working in jobs that require face-to-face interactions [10].

Labour market outcomes varied considerably across the five countries during the pandemic. Egypt and Tunisia recorded the highest incidence of temporary layoffs reductions in working hours and incomes. Krafft et al. [25] also found that Morocco experienced increasing unemployment rates and declining labour force participation during the pandemic compared to other Arab countries that have gradually improved labour market conditions over time. The differences between countries are mainly due to the various policies adopted to buffer the negative impact of COVID-19 on the labour market. Therefore, we highly recommend that future studies investigate the impact of country-level policies (financial, monetary, and regulatory policies) on the status of Arab working women during COVID-19.

Improving women's working conditions and supporting those who have suffered negative consequences during the pandemic is a critical matter to enhance their long-term well-being. To narrow the gender gap in labour market outcomes, policymakers should ensure reliable and affordable childcare options, allow women to work remotely, and provide flexible working hours. Shifting towards working at home could be an endowment for women to continue working. Family leave should also be allowed for both men and women to integrate their family roles. Encouraging equal allocation of childcare between parents during crises would induce long-term changes in inherited attitudes toward paternal responsibilities and the work environment. In addition, special social protection schemes should be dedicated to working women, especially in the informal sector. Like other countries, MENA countries should adopt job retention policies and income replacement schemes by introducing fiscal stimulus packages that reduce labour costs in affected companies, offset lost incomes and help companies retain their employees.

This study makes several contributions. First, it adds to the limited studies that examined the gender gap in labour market outcomes during the COVID-19 pandemic, providing new evidence of gender inequality in the understudied MENA region. Second, unlike previous studies that were limited to modeling the bivariate outcomes, our study used MPM to estimate the determinants of labour market outcomes simultaneously. Third, the study highlights the effect of the increased childcare and housework responsibilities during the pandemic in shaping women's labour market outcomes. Finally, the study also shed light on the importance of telework availability in sheltering women from the negative impacts of COVID-19 and its related restrictions. However, the study had some limitations. First, the data represents mobile phone owners 18–64 years old. Thus, the data is not nationally representative, and some population segments are expected to be missed including the less educated, the poor, and individuals without mobile phones. Second, the analysis did not control some factors that could drive employment outcomes such as the necessity of physical proximity, the share of tasks that can be done from home, and the amount of demand for different industries.

## 5 Conclusion

COVID-19 has caused profound effects on the labour market, resulting in adverse implications for gender equality. The study focused on assessing the economic repercussions of the COVID-19 crisis from a gender perspective in five countries in the MENA region. Despite the prevalence of multiple adverse work outcomes during the pandemic, no prior investigation considered and contrasted all these outcomes by gender over the countries we analyzed. Response variables comprised reporting five negative labour outcomes: job loss (temporarily or permanently), reduced working hours, decreased income, and change in the main job during the pandemic. We used MPM to estimate jointly the association between gender and multiple labour outcomes while controlling other covariates and correlations among labour outcomes. MPM has an advance over traditional approaches because of considering correlation between labour outcomes and allowing for more efficient estimates. The study provides new evidence of gender inequality in the understudied MENA region. Women were more likely to be permanently laid off and change their main activity than males. The results documented that increased childcare and housework responsibilities have negatively affected women's work during the pandemic. Higher socioeconomic status played a significant role in alleviating the harmful impacts of the crisis; having a college degree and being in the highest income quartile served as protective factors against experiencing negative labour outcomes. Telework availability has succeeded in protecting women from the negative effects of COVID-19. Supportive policies should be introduced for women to reduce inequality in job losses. Working from home represents a turning point in work alternatives that can be adapted to support women's work, especially those more amenable to remote work due to maternity tasks. Future studies could investigate the impact of country-level policies (financial, monetary, and regulatory policies) on the status of Arab working women during COVID-19.

### Ethics approval

Not applicable.

### Consent for publication

Not applicable.

## Author contribution statement

Suzan Abdel-Rahman: Performed the experiments; Contributed reagents, materials, analysis tools or data; Wrote the paper.

Fuad A. Awwad: Conceived and designed the experiments; Analyzed and interpreted the data.

Muhammad Qasim: Analyzed and interpreted the data.

Mohamed R. Abonazel: Analyzed and interpreted the data; Contributed reagents, materials, analysis tools or data.

## Funding statement

This project is funded by 10.13039/501100002383King Saud University, Riyadh, Saudi Arabia.

## Data availability statement

The authors do not have permission to share data.

## Additional information

No additional information is available for this paper.

## Declaration of competing interest

We hereby declare that this manuscript is original and there is no competing interest.
